# 8-{[3-(3-Meth­oxy­phen­yl)-1,2,4-oxa­diazol-5-yl]meth­oxy}quinoline monohydrate

**DOI:** 10.1107/S1600536813010271

**Published:** 2013-04-20

**Authors:** Hong Shen, Shu-Yuan Bai, Xin-Yi Han, Xiang-Zhi Li, Hai-Bo Wang

**Affiliations:** a College of Food Science and Light Industry, Nanjing University of Technology, Xinmofan Road No. 5 Nanjing, Nanjing 210009, People’s Republic of China; b College of Science, Nanjing University of Technology, Xinmofan Road No. 5 Nanjing, Nanjing 210009, People’s Republic of China

## Abstract

In the title hydrate, C_19_H_15_N_3_O_3_·H_2_O, the three aromatic groups in the quinoline derivative are close to coplanar: the central oxa­diazole fragment makes dihedral angles of 15.7 (2)° with the benzene ring and 5.30 (14)° with the quinoline ring system. In the crystal, the organic mol­ecules are connected with water mol­ecules by pairs of O—H⋯N hydrogen bonds involving the quinoline and oxa­diazole N atoms. The mol­ecules form stacks along the *a* axis, neighboring mol­ecules within each stack being related by inversion and the shortest distance between the centroids of the oxa­diazole and pyridine rings being 3.500 (2) Å. Mol­ecules from neighboring stacks are linked by weak C—H⋯O hydrogen bonds, forming a three-dimensional structure.

## Related literature

For the preparation of the title compound, see: Shishue & Henry (1989 ▶). For the general synthetic procedure, see: Munoz-Muniz & Juaristi (2003 ▶). For standard bond-length data, see: Allen *et al.* (1987 ▶).
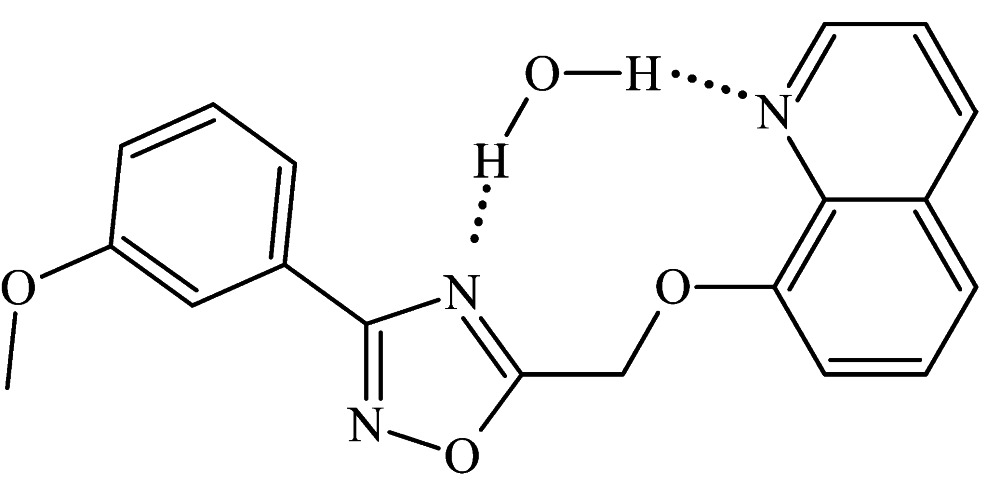


## Experimental

### 

#### Crystal data

C_19_H_15_N_3_O_3_·H_2_O
*M* *_r_* = 351.36
Monoclinic, 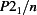

*a* = 7.9510 (16) Å
*b* = 6.9870 (14) Å
*c* = 30.395 (6) Å
β = 92.31 (3)°
*V* = 1687.2 (6) Å^3^
*Z* = 4
Mo *K*α radiation
μ = 0.10 mm^−1^
*T* = 293 K
0.30 × 0.20 × 0.10 mm

#### Data collection

Enraf–Nonius CAD-4 diffractometer
Absorption correction: ψ scan (North *et al.*, 1968 ▶) *T*_min_ = 0.971, *T*_max_ = 0.990
3339 measured reflections
3101 independent reflections
2183 reflections with *I* > 2σ(*I*)
*R*_int_ = 0.056
3 standard reflections every 200 reflections intensity decay: 1%

#### Refinement

*R*[*F* ^2^ > 2σ(*F* ^2^)] = 0.055
*wR*(*F* ^2^) = 0.195
*S* = 1.01
3101 reflections
244 parameters
1 restraint
H atoms treated by a mixture of independent and constrained refinement
Δρ_max_ = 0.22 e Å^−3^
Δρ_min_ = −0.21 e Å^−3^

### 

Data collection: *CAD-4 EXPRESS* (Enraf–Nonius, 1994 ▶); cell refinement: *CAD-4 EXPRESS*; data reduction: *XCAD4* (Harms & Wocadlo,1995 ▶); program(s) used to solve structure: *SHELXS97* (Sheldrick, 2008 ▶); program(s) used to refine structure: *SHELXL97* (Sheldrick, 2008 ▶); molecular graphics: *PLATON* (Spek, 2009 ▶); software used to prepare material for publication: *PLATON*.

## Supplementary Material

Crystal structure: contains datablock(s) global, I. DOI: 10.1107/S1600536813010271/yk2090sup1.cif

Structure factors: contains datablock(s) I. DOI: 10.1107/S1600536813010271/yk2090Isup2.hkl

Supplementary material file. DOI: 10.1107/S1600536813010271/yk2090Isup3.cml

Additional supplementary materials:  crystallographic information; 3D view; checkCIF report

## Figures and Tables

**Table 1:** Hydrogen-bond geometry (Å, °)

*D*—H⋯*A*	*D*—H	H⋯*A*	*D*⋯*A*	*D*—H⋯*A*
O*W*—H*WB*⋯N2	0.92 (5)	2.05 (5)	2.965 (3)	174 (5)
O*W*—H*WA*⋯N3	0.88 (4)	1.96 (4)	2.840 (4)	172 (4)
C10—H10*A*⋯O*W* ^i^	0.97	2.39	3.340 (4)	165
C16—H16*A*⋯O1^ii^	0.93	2.58	3.309 (4)	135

Symmetry codes: (i) 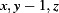; (ii) 
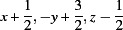
.

**Fig. 1.**
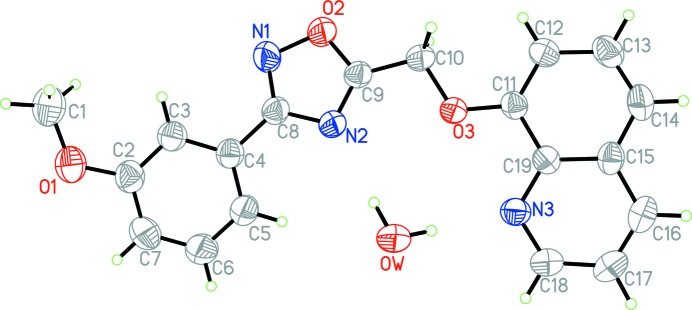
The molecular structure of the title compound with the atom-numbering scheme. Displacement ellipsoids are drawn at the 50% probability level.

**Fig. 2.**
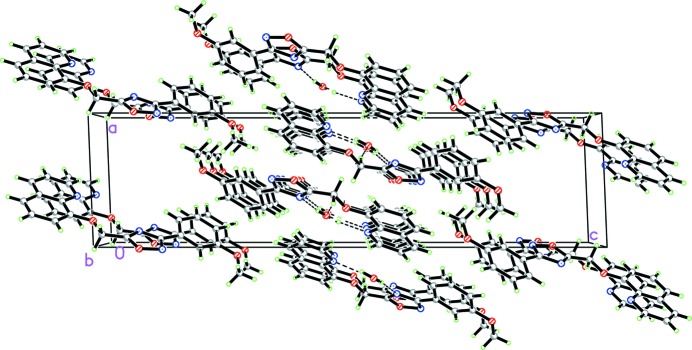
A packing diagram of the title compound viewed down the c axis. Dashed lines indicate intermolecular C—H···O interactions.

## supplementary crystallographic information

### Comment

1,2,4-oxadiazole derivatives plays an important role in medicine and as pesticides.They show high biological activity, such as antibacterial, anti-hiv and weed control. The 1,2,4-oxadiazole derivatives also can be used in metal-ions fluorescent recognition. The title compound, 8-{[3-(3-methoxyphenyl)-1,2,4-oxadiazol-5-yl]methoxy}quinoline, was used in metal-ions fluorescent recognition. In the molecule of 8-{[3-(3-methoxyphenyl)-1,2,4-oxadiazol-5-yl]methoxy} quinoline monohydrate (Fig,1), the bond length (Allen *et al.*, 1987) and angles are within normal ranges. The molecule is almost planar. In the crystal, the intermolecular C16—H16···O1 hydrogen bonds link the molecules into zig-zag chains along the *c* axis and the intermolecular C10-H10···OW hydrogen bonds link the molecules into zig-zag chains along the *b* axis, thus forming a stable structure (Fig. 2).

### Experimental

The title compound, 8-{[3-(3-methoxyphenyl)-1,2,4-oxadiazol-5-yl]methoxy} quinoline was prepared by the literature method (Shishue & Henry, 1989). 3-(4-Methoxy-phenyl)-5-chloromethyl-1,2,4-oxadizole(1.6 g, 8.2 mmol), 8-hydroxyquinoline(1.2 g, 8.2 mmol), potassium carbonate(1.7 g, 12.3 mmol) and potassium iodide (catalytic amount) were added to acetone (20 ml), and then the mixture was heated to reflux for 6 hours, cooled to room temperature, filtered and evaporated to afford the yellow solid. The crude product was recrystallized from ethyl acetate. Yield 2 g (80.5%). Crystals suitable for X-ray analysis were obtained by slow evaporation of an ethanol solution.

### Refinement

H atoms were positioned geometrically, with C—H = 0.93, 0.97 and 0.96 Å for aromatic, methylene and methyl H, respectively, and constrained to ride on their parent atoms, with U_iso_(H) = xU_eq_(C,N), where x = 1.5 for methyl H and x = 1.2 for all other H atoms. In the absence of significant anomalous dispersion effects, 1739 Friedel pairs were merged.

### Crystal data

**Table:**

C_19_H_15_N_3_O_3_·H_2_O	*F*(000) = 736
*M**_r_* = 351.36	*D*_x_ = 1.383 Mg m^−^^3^
Monoclinic, *P*2_1_/*n*	Melting point: 338 K
Hall symbol: -P 2yn	Mo *K*α radiation, λ = 0.71073 Å
*a* = 7.9510 (16) Å	Cell parameters from 25 reflections
*b* = 6.9870 (14) Å	θ = 10–14°
*c* = 30.395 (6) Å	µ = 0.10 mm^−^^1^
β = 92.31 (3)°	*T* = 293 K
*V* = 1687.2 (6) Å^3^	Block, colourless
*Z* = 4	0.30 × 0.20 × 0.10 mm

### Data collection

**Table:**

Enraf–Nonius CAD-4 diffractometer	2183 reflections with *I* > 2σ(*I*)
Radiation source: fine-focus sealed tube	*R*_int_ = 0.056
Graphite monochromator	θ_max_ = 25.4°, θ_min_ = 1.3°
ω/2θ scans	*h* = 0→9
Absorption correction: ψ scan (North *et al.*, 1968)	*k* = 0→8
*T*_min_ = 0.971, *T*_max_ = 0.990	*l* = −36→36
3339 measured reflections	3 standard reflections every 200 reflections
3101 independent reflections	intensity decay: 1%

### Refinement

**Table:**

Refinement on *F*^2^	Secondary atom site location: difference Fourier map
Least-squares matrix: full	Hydrogen site location: inferred from neighbouring sites
*R*[*F*^2^ > 2σ(*F*^2^)] = 0.055	H atoms treated by a mixture of independent and constrained refinement
*wR*(*F*^2^) = 0.195	*w* = 1/[σ^2^(*F*_o_^2^) + (0.1*P*)^2^ + 1.4*P*] where *P* = (*F*_o_^2^ + 2*F*_c_^2^)/3
*S* = 1.01	(Δ/σ)_max_ < 0.001
3101 reflections	Δρ_max_ = 0.22 e Å^−^^3^
244 parameters	Δρ_min_ = −0.21 e Å^−^^3^
1 restraint	Extinction correction: *SHELXL97* (Sheldrick, 2008), Fc^*^=kFc[1+0.001xFc^2^λ^3^/sin(2θ)]^-1/4^
Primary atom site location: structure-invariant direct methods	Extinction coefficient: 0.045 (4)

### Special details

**Table:**

Geometry. All esds (except the esd in the dihedral angle between two l.s. planes) are estimated using the full covariance matrix. The cell esds are taken into account individually in the estimation of esds in distances, angles and torsion angles; correlations between esds in cell parameters are only used when they are defined by crystal symmetry. An approximate (isotropic) treatment of cell esds is used for estimating esds involving l.s. planes.
Refinement. Refinement of F^2^ against ALL reflections. The weighted R-factor wR and goodness of fit S are based on F^2^, conventional R-factors R are based on F, with F set to zero for negative F^2^. The threshold expression of F^2^ > 2sigma(F^2^) is used only for calculating R-factors(gt) etc. and is not relevant to the choice of reflections for refinement. R-factors based on F^2^ are statistically about twice as large as those based on F, and R- factors based on ALL data will be even larger.

### Fractional atomic coordinates and isotropic or equivalent isotropic displacement parameters (Å^2^)

**Table:**

	*x*	*y*	*z*	*U*_iso_*/*U*_eq_	
O1	0.4276 (3)	0.7480 (4)	0.77526 (8)	0.0734 (8)	
N1	0.4925 (4)	0.3104 (4)	0.63924 (9)	0.0614 (8)	
C1	0.3027 (6)	0.6086 (7)	0.78108 (13)	0.0851 (13)	
H1B	0.2535	0.6270	0.8091	0.128*	
H1C	0.3522	0.4834	0.7801	0.128*	
H1D	0.2171	0.6201	0.7580	0.128*	
N2	0.6199 (3)	0.4981 (3)	0.59050 (8)	0.0431 (6)	
O2	0.5011 (3)	0.2177 (3)	0.59795 (8)	0.0607 (7)	
C2	0.5139 (4)	0.7451 (5)	0.73722 (10)	0.0550 (8)	
O3	0.6989 (3)	0.4102 (3)	0.50481 (6)	0.0470 (6)	
N3	0.8809 (3)	0.6747 (3)	0.46505 (8)	0.0454 (6)	
C3	0.4933 (4)	0.6079 (5)	0.70490 (10)	0.0504 (8)	
H3B	0.4178	0.5078	0.7083	0.061*	
C4	0.5858 (4)	0.6199 (4)	0.66719 (9)	0.0464 (7)	
C5	0.6971 (4)	0.7703 (5)	0.66207 (11)	0.0595 (9)	
H5A	0.7580	0.7799	0.6367	0.071*	
C6	0.7167 (5)	0.9053 (6)	0.69492 (12)	0.0703 (11)	
H6A	0.7922	1.0056	0.6917	0.084*	
C7	0.6265 (5)	0.8940 (5)	0.73233 (12)	0.0658 (10)	
H7A	0.6409	0.9859	0.7543	0.079*	
C8	0.5642 (4)	0.4745 (4)	0.63264 (10)	0.0442 (7)	
C9	0.5784 (4)	0.3397 (4)	0.57156 (9)	0.0416 (7)	
C10	0.6032 (4)	0.2704 (4)	0.52647 (10)	0.0451 (7)	
H10A	0.6624	0.1490	0.5273	0.054*	
H10B	0.4953	0.2523	0.5110	0.054*	
C11	0.7455 (3)	0.3672 (4)	0.46310 (9)	0.0400 (7)	
C12	0.7012 (4)	0.2037 (4)	0.44091 (10)	0.0489 (8)	
H12A	0.6377	0.1106	0.4545	0.059*	
C13	0.7514 (4)	0.1761 (5)	0.39758 (11)	0.0551 (8)	
H13A	0.7193	0.0650	0.3826	0.066*	
C14	0.8448 (4)	0.3072 (5)	0.37739 (10)	0.0542 (8)	
H14A	0.8781	0.2850	0.3489	0.065*	
C15	0.8928 (4)	0.4787 (4)	0.39913 (9)	0.0455 (7)	
C16	0.9866 (4)	0.6257 (5)	0.37967 (11)	0.0558 (9)	
H16A	1.0215	0.6119	0.3510	0.067*	
C17	1.0257 (4)	0.7863 (5)	0.40267 (12)	0.0583 (9)	
H17A	1.0888	0.8829	0.3903	0.070*	
C18	0.9697 (4)	0.8045 (5)	0.44528 (11)	0.0523 (8)	
H18A	0.9970	0.9159	0.4607	0.063*	
C19	0.8423 (3)	0.5107 (4)	0.44272 (9)	0.0402 (7)	
OW	0.7589 (3)	0.8317 (3)	0.54382 (9)	0.0647 (7)	
HWB	0.724 (6)	0.727 (7)	0.5591 (16)	0.106 (16)*	
HWA	0.806 (6)	0.781 (7)	0.5207 (12)	0.110 (18)*	

### Atomic displacement parameters (Å^2^)

**Table:**

	*U*^11^	*U*^22^	*U*^33^	*U*^12^	*U*^13^	*U*^23^
O1	0.0923 (18)	0.0818 (18)	0.0472 (14)	−0.0117 (15)	0.0183 (12)	−0.0116 (13)
N1	0.090 (2)	0.0494 (16)	0.0465 (15)	−0.0100 (15)	0.0192 (14)	−0.0026 (13)
C1	0.107 (3)	0.095 (3)	0.056 (2)	−0.014 (3)	0.030 (2)	−0.006 (2)
N2	0.0485 (14)	0.0388 (13)	0.0425 (13)	−0.0023 (11)	0.0059 (10)	0.0021 (11)
O2	0.0865 (16)	0.0423 (12)	0.0550 (14)	−0.0139 (11)	0.0220 (12)	−0.0013 (10)
C2	0.062 (2)	0.060 (2)	0.0432 (17)	0.0007 (17)	0.0033 (14)	−0.0004 (15)
O3	0.0630 (13)	0.0356 (11)	0.0433 (11)	−0.0086 (9)	0.0121 (9)	−0.0049 (9)
N3	0.0484 (14)	0.0391 (13)	0.0484 (14)	−0.0026 (11)	0.0004 (11)	−0.0006 (11)
C3	0.0566 (18)	0.0511 (18)	0.0436 (17)	−0.0030 (15)	0.0020 (14)	0.0021 (14)
C4	0.0487 (16)	0.0486 (17)	0.0420 (16)	0.0013 (14)	0.0019 (13)	0.0020 (13)
C5	0.064 (2)	0.065 (2)	0.0500 (19)	−0.0137 (17)	0.0110 (15)	−0.0045 (17)
C6	0.078 (2)	0.071 (2)	0.062 (2)	−0.025 (2)	0.0082 (19)	−0.0123 (19)
C7	0.079 (2)	0.066 (2)	0.052 (2)	−0.008 (2)	0.0004 (17)	−0.0162 (18)
C8	0.0482 (16)	0.0408 (16)	0.0438 (16)	0.0005 (13)	0.0054 (12)	0.0041 (13)
C9	0.0467 (16)	0.0349 (14)	0.0435 (16)	0.0004 (12)	0.0040 (12)	0.0042 (13)
C10	0.0527 (17)	0.0358 (15)	0.0470 (17)	−0.0047 (13)	0.0053 (13)	0.0022 (13)
C11	0.0454 (15)	0.0353 (14)	0.0393 (15)	0.0046 (12)	0.0021 (12)	−0.0023 (12)
C12	0.0566 (18)	0.0401 (16)	0.0500 (18)	−0.0026 (14)	0.0030 (14)	−0.0054 (14)
C13	0.066 (2)	0.0465 (18)	0.0525 (19)	0.0008 (16)	0.0010 (15)	−0.0142 (16)
C14	0.065 (2)	0.060 (2)	0.0372 (16)	0.0088 (17)	0.0026 (14)	−0.0049 (15)
C15	0.0490 (16)	0.0491 (17)	0.0382 (15)	0.0085 (14)	0.0010 (12)	0.0037 (13)
C16	0.0588 (19)	0.065 (2)	0.0443 (17)	0.0067 (16)	0.0079 (14)	0.0122 (16)
C17	0.063 (2)	0.0495 (19)	0.063 (2)	−0.0027 (16)	0.0103 (16)	0.0154 (17)
C18	0.0580 (19)	0.0410 (17)	0.0578 (19)	−0.0060 (15)	0.0032 (15)	0.0008 (15)
C19	0.0427 (15)	0.0378 (15)	0.0397 (15)	0.0039 (12)	−0.0027 (12)	−0.0003 (12)
OW	0.0898 (19)	0.0395 (12)	0.0661 (17)	−0.0104 (13)	0.0176 (14)	−0.0020 (12)

### Geometric parameters (Å, º)

**Table:**

O1—C2	1.368 (4)	C6—H6A	0.9300
O1—C1	1.408 (5)	C7—H7A	0.9300
N1—C8	1.300 (4)	C9—C10	1.474 (4)
N1—O2	1.416 (3)	C10—H10A	0.9700
C1—H1B	0.9600	C10—H10B	0.9700
C1—H1C	0.9600	C11—C12	1.366 (4)
C1—H1D	0.9600	C11—C19	1.421 (4)
N2—C9	1.285 (4)	C12—C13	1.405 (4)
N2—C8	1.382 (4)	C12—H12A	0.9300
O2—C9	1.337 (3)	C13—C14	1.343 (5)
C2—C3	1.378 (4)	C13—H13A	0.9300
C2—C7	1.385 (5)	C14—C15	1.413 (4)
O3—C11	1.368 (3)	C14—H14A	0.9300
O3—C10	1.417 (3)	C15—C16	1.414 (4)
N3—C18	1.310 (4)	C15—C19	1.417 (4)
N3—C19	1.360 (4)	C16—C17	1.352 (5)
C3—C4	1.389 (4)	C16—H16A	0.9300
C3—H3B	0.9300	C17—C18	1.392 (5)
C4—C5	1.386 (4)	C17—H17A	0.9300
C4—C8	1.466 (4)	C18—H18A	0.9300
C5—C6	1.378 (5)	OW—HWB	0.92 (5)
C5—H5A	0.9300	OW—HWA	0.881 (19)
C6—C7	1.371 (5)		
			
C2—O1—C1	118.5 (3)	O2—C9—C10	115.5 (2)
C8—N1—O2	103.3 (2)	O3—C10—C9	107.5 (2)
O1—C1—H1B	109.5	O3—C10—H10A	110.2
O1—C1—H1C	109.5	C9—C10—H10A	110.2
H1B—C1—H1C	109.5	O3—C10—H10B	110.2
O1—C1—H1D	109.5	C9—C10—H10B	110.2
H1B—C1—H1D	109.5	H10A—C10—H10B	108.5
H1C—C1—H1D	109.5	C12—C11—O3	124.5 (3)
C9—N2—C8	103.1 (2)	C12—C11—C19	120.5 (3)
C9—O2—N1	106.3 (2)	O3—C11—C19	114.9 (2)
O1—C2—C3	124.3 (3)	C11—C12—C13	120.1 (3)
O1—C2—C7	115.3 (3)	C11—C12—H12A	120.0
C3—C2—C7	120.4 (3)	C13—C12—H12A	120.0
C11—O3—C10	116.7 (2)	C14—C13—C12	121.2 (3)
C18—N3—C19	118.0 (3)	C14—C13—H13A	119.4
C2—C3—C4	119.6 (3)	C12—C13—H13A	119.4
C2—C3—H3B	120.2	C13—C14—C15	120.6 (3)
C4—C3—H3B	120.2	C13—C14—H14A	119.7
C5—C4—C3	120.0 (3)	C15—C14—H14A	119.7
C5—C4—C8	120.1 (3)	C14—C15—C16	123.9 (3)
C3—C4—C8	119.9 (3)	C14—C15—C19	119.3 (3)
C6—C5—C4	119.4 (3)	C16—C15—C19	116.8 (3)
C6—C5—H5A	120.3	C17—C16—C15	120.1 (3)
C4—C5—H5A	120.3	C17—C16—H16A	120.0
C7—C6—C5	120.9 (3)	C15—C16—H16A	120.0
C7—C6—H6A	119.5	C16—C17—C18	118.8 (3)
C5—C6—H6A	119.5	C16—C17—H17A	120.6
C6—C7—C2	119.6 (3)	C18—C17—H17A	120.6
C6—C7—H7A	120.2	N3—C18—C17	124.2 (3)
C2—C7—H7A	120.2	N3—C18—H18A	117.9
N1—C8—N2	114.0 (3)	C17—C18—H18A	117.9
N1—C8—C4	122.7 (3)	N3—C19—C15	122.2 (3)
N2—C8—C4	123.3 (3)	N3—C19—C11	119.4 (2)
N2—C9—O2	113.3 (3)	C15—C19—C11	118.3 (3)
N2—C9—C10	131.2 (3)	HWB—OW—HWA	103 (4)
			
C8—N1—O2—C9	0.2 (3)	N2—C9—C10—O3	4.2 (4)
C1—O1—C2—C3	−3.7 (5)	O2—C9—C10—O3	−175.0 (2)
C1—O1—C2—C7	175.7 (4)	C10—O3—C11—C12	2.4 (4)
O1—C2—C3—C4	179.1 (3)	C10—O3—C11—C19	−179.6 (2)
C7—C2—C3—C4	−0.2 (5)	O3—C11—C12—C13	178.0 (3)
C2—C3—C4—C5	−0.6 (5)	C19—C11—C12—C13	0.1 (4)
C2—C3—C4—C8	−179.9 (3)	C11—C12—C13—C14	0.8 (5)
C3—C4—C5—C6	1.0 (5)	C12—C13—C14—C15	−1.1 (5)
C8—C4—C5—C6	−179.7 (3)	C13—C14—C15—C16	−178.1 (3)
C4—C5—C6—C7	−0.7 (6)	C13—C14—C15—C19	0.4 (5)
C5—C6—C7—C2	−0.1 (6)	C14—C15—C16—C17	179.3 (3)
O1—C2—C7—C6	−178.8 (4)	C19—C15—C16—C17	0.7 (4)
C3—C2—C7—C6	0.5 (6)	C15—C16—C17—C18	−1.0 (5)
O2—N1—C8—N2	−0.2 (4)	C19—N3—C18—C17	0.7 (5)
O2—N1—C8—C4	−179.3 (3)	C16—C17—C18—N3	0.3 (5)
C9—N2—C8—N1	0.1 (3)	C18—N3—C19—C15	−1.0 (4)
C9—N2—C8—C4	179.2 (3)	C18—N3—C19—C11	−179.7 (3)
C5—C4—C8—N1	164.3 (3)	C14—C15—C19—N3	−178.4 (3)
C3—C4—C8—N1	−16.4 (5)	C16—C15—C19—N3	0.3 (4)
C5—C4—C8—N2	−14.8 (5)	C14—C15—C19—C11	0.4 (4)
C3—C4—C8—N2	164.6 (3)	C16—C15—C19—C11	179.0 (3)
C8—N2—C9—O2	0.1 (3)	C12—C11—C19—N3	178.2 (3)
C8—N2—C9—C10	−179.2 (3)	O3—C11—C19—N3	0.0 (4)
N1—O2—C9—N2	−0.2 (3)	C12—C11—C19—C15	−0.7 (4)
N1—O2—C9—C10	179.2 (3)	O3—C11—C19—C15	−178.8 (2)
C11—O3—C10—C9	175.8 (2)		

### Hydrogen-bond geometry (Å, º)

**Table:**

*D*—H···*A*	*D*—H	H···*A*	*D*···*A*	*D*—H···*A*
O*W*—H*WB*···N2	0.92 (5)	2.05 (5)	2.965 (3)	174 (5)
O*W*—H*WA*···N3	0.88 (4)	1.96 (4)	2.840 (4)	172 (4)
C10—H10*A*···O*W*^i^	0.97	2.39	3.340 (4)	165
C16—H16*A*···O1^ii^	0.93	2.58	3.309 (4)	135

Symmetry codes: (i) *x*, *y*−1, *z*; (ii) *x*+1/2, −*y*+3/2, *z*−1/2.

## References

[bb1] Allen, F. H., Kennard, O., Watson, D. G., Brammer, L., Orpen, A. G. & Taylor, R. (1987). *J. Chem. Soc. Perkin Trans. 2*, pp. S1–19.

[bb2] Enraf–Nonius (1994). *CAD-4 EXPRESS* Enraf–Nonius, Delft. The Netherlands.

[bb3] Harms, K. & Wocadlo, S. (1995). *XCAD4* University of Marburg, Germany.

[bb4] Munoz-Muniz, O. & Juaristi, E. (2003). *Tetrahedron*, **59**, 4223–4229.

[bb5] North, A. C. T., Phillips, D. C. & Mathews, F. S. (1968). *Acta Cryst.* A**24**, 351–359.

[bb6] Sheldrick, G. M. (2008). *Acta Cryst.* A**64**, 112–122. 10.1107/S0108767307043930 18156677

[bb7] Shishue, C. & Henry, J. S. (1989). *J. Heterocycl. Chem.*, **26**, 125–128.

[bb8] Spek, A. L. (2009). *Acta Cryst.* D**65**, 148–155. 10.1107/S090744490804362X PMC2631630 19171970

